# Aberrant Development and Synaptic Transmission of Cerebellar Cortex in a VPA Induced Mouse Autism Model

**DOI:** 10.3389/fncel.2018.00500

**Published:** 2018-12-21

**Authors:** Ruanna Wang, Jiahui Tan, Junxiu Guo, Yuhan Zheng, Qing Han, Kwok-Fai So, Jiandong Yu, Li Zhang

**Affiliations:** Joint International Research Laboratory of CNS Regeneration, Guangdong-Hong Kong-Macau Institute of CNS Regeneration, Jinan University, Guangzhou, China

**Keywords:** autism, cerebellum, environmental exposure, motor learning, postnatal development

## Abstract

Autistic spectral disorder (ASD) is a prevalent neurodevelopmental disease that affects multiple brain regions. Both clinical and animal studies have revealed the possible involvement of the cerebellum in ASD pathology. In this study, we generated a rodent ASD model through a single prenatal administration of valproic acid (VPA) into pregnant mice, followed by cerebellar morphological and functional studies of the offspring. Behavioral studies showed that VPA exposure led to retardation of critical motor reflexes in juveniles and impaired learning in a tone-conditioned complex motor task in adults. These behavioral phenotypes were associated with premature migration and excess apoptosis of the granular cell (GC) precursor in the cerebellar cortex during the early postnatal period, and the decreased cell density and impaired dendritic arborization of the Purkinje neurons. On acute cerebellar slices, suppressed synaptic transmission of the Purkinje cells were reported in the VPA-treated mice. In summary, converging evidence from anatomical, electrophysiological and behavioral abnormalities in the VPA-treated mice suggest cerebellar pathology in ASD and indicate the potential values of motor dysfunction in the early diagnosis of ASD.

## Introduction

The cerebellum has been recognized as the brain region mediating fine motor coordination and complex motor skill learning. Recent evidence however, has revealed the role of the cerebellum in psychiatric disorders including major depressive disorder (Su et al., 2014), schizophrenia (Mothersill et al., 2016) and autistic spectral disorder (ASD; Fatemi et al., 2012; Wang et al., 2014). Postmortem examinations of ASD patient brains reported cerebellar neuropathology (Hampson and Blatt, 2015). In particular, ASD children frequently present cerebellar associated motor disorders (McPhillips et al., 2014; Mosconi et al., 2015), which can occur at early age before the onset of language or social deficits (Lloyd et al., 2013). Currently, the potential value of motor dysfunction in the early diagnosis of ASD is being continuously discussed (Whyatt and Craig, 2013; Zwaigenbaum et al., 2013). It is thus necessary to further elaborate the cerebellar neuropathology associated with ASD.

ASD etiology can be attributed to genetic mutations and/or environmental exposures. Among the known ASD risk genes, *Pten* (Cupolillo et al., 2016) or *Shank2* (Peter et al., 2016) mutations in the cerebellar Purkinje cells lead to impaired cell morphology or synaptic transmission, which are associated with motor learning and social deficits. These mouse studies support the involvement of the cerebellum in ASD pathogenesis. An alternative group of ASD risk factors consists of prenatal exposures of specific chemicals or drugs (Mandy and Lai, 2016). Valproic acid (VPA) is one anti-seizure drug and has been reported to dramatically elevate ASD risk in offspring (Ornoy, 2009; Roullet et al., 2013). Prenatal VPA exposure in rodents can replicate ASD-like symptoms including repetitive behaviors and social deficits (Markram et al., 2008; Yochum et al., 2008). Therefore, VPA overdosage has become one widely accepted model to elaborate the neurobiological mechanism of ASD (Nicolini and Fahnestock, 2018). The neurodevelopmental effects of VPA cover multiple brain regions including the prefrontal cortex (Codagnone et al., 2015), hippocampus (Bristot Silvestrin et al., 2013) and amygdala (Sosa-Díaz et al., 2014). In the cerebellum, previous studies have revealed an enhanced apoptosis in the external granular layer (EGL) after early postnatal VPA exposure in mice (Yochum et al., 2008) or rats (Kim et al., 2013). However, the effects of prenatal VPA exposure on the cerebellar development, or its correlation with motor deficits has not been reported yet.

In the current study, we utilized a single VPA injection in pregnant mice at embryonic day 10.5 (E10.5) and found that VPA exposure led to the retardation of motor reflexes in juveniles as well as deficits in complex motor learning tasks in adults. Those behavioral phenotypes were associated with premature migration and elevated apoptosis of granular cell (GC) precursors in the cerebellum during the first two postnatal weeks. The VPA-treated mice also had a decreased cell density and an impaired dendritic arborization of the cerebellar Purkinje neurons in both the juvenile and adult stages. Such structural deformation of the cerebellar cortex was accompanied with impaired excitatory and inhibitory synaptic transmission. In sum, our results describe both the structural and functional impairments of the cerebellum caused by prenatal VPA exposure, further elaborating the potential linkage between cerebellar pathology and ASD.

## Materials and Methods

### Experimental Animals

Female C57BL/6 mice (7–8 weeks old) were mated with male mice at 8 pm. The vaginal plug was examined at 8 am the following morning. Those females having plugs were singly housed and denoted as embryonic at day 0.5 (E0.5). VPA (500 mg/kg body weight, in sodium salt, Sigma, Ronkonkoma, NY, USA) was prepared in 50 mg/mL sterile saline and injected into the peritoneal cavity of pregnant mice at E10.5. The control group received 0.1 mL sterile saline. Male offspring were used for further assays. The animal protocol was approved by the Jinan University Institutional Animal Care and Use Committee.

### Developmental Examination

To characterize postnatal developmental patterns of mice, we utilized a test battery of developmental milestones as previously described (Zhang et al., 2014). Briefly speaking, juvenile mice from both VPA-treated and saline-treated groups were examined for different neural reflex and body development markers, including cliff avoidance (the avoidance behavior when the mouse’ forepaws were approaching the edge of one platform), grasping reflex (to grasp one small metal bar with the forepaws), negative geotaxis (to recover from a head-down position on an inclined plane), surface righting (turning its body around from an upside-down position), air righting (recovering a normal prone position when releasing from the height in a supine position), bar holding (ability to hold a metal bar with the forepaws for more than 5 s), pinnae detachment and eye opening. The day of onset was recorded, and any positive reflexes were confirmed the following day. We examined two cerebellar associated motor reflexes: (1) negative geotaxis: the mouse was placed on a 30° inclined plane with its head facing downwards. The positive reflex was identified when the mouse could turn its body around to a head-up position within 30 s; and (2) air righting reflex: the mouse was released from a 30 cm height using an inverted supine position (with its belly facing upwards). The positive reflex was identified when the mouse could recover the normal prone position (belly facing downwards against the soft bedding) when landing.

### Mouse Behavioral Assays

To validate the ASD phenotype of the VPA model, the 3-chamber sociability assay was employed first, as previously described (Peter et al., 2016). In brief, the mouse was first placed into the central zone to freely explore the three chambers (divided from a 60 cm × 45 cm clear box) for 15 min. During the second 15-min stage, one age-matched male mouse (S1) was placed into one chamber within a round wire cage. The time for the test mouse to explore and interact with the S1 mouse was recorded. For the third stage, a second stranger mouse (S2) was introduced to the other side of the chamber and the interaction time with both the S1 and S2 mice, was recorded during the 15-min session.

For the open field assay, a clear box (50 cm × 50 cm) was utilized to measure the locomotor activity of mice in a single 15 min session, during which the total distance of each mouse was analyzed.

The accelerating Rota-rod test was performed as previously described (Zhang et al., 2014). The mouse was trained on the rod in six consecutive daily sessions, with 5 rpm initial velocity and 80 rpm maximal at 5 min.

**Figure 1:**
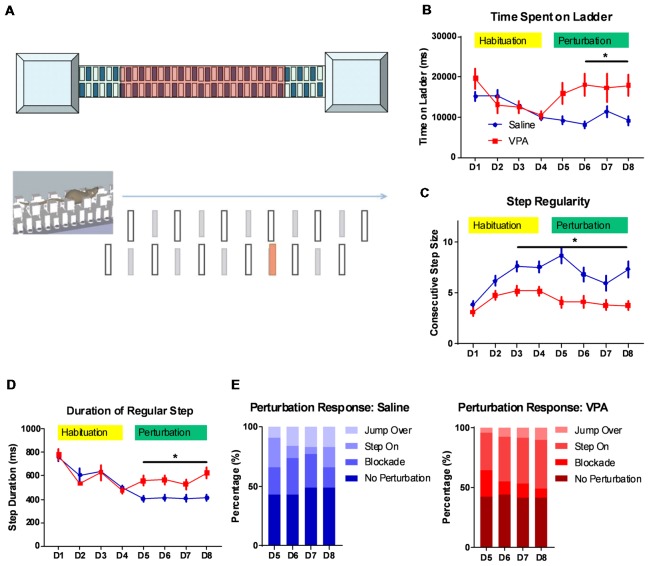
Impaired complex motor learning in valproic acid (VPA)-treated mice.** (A)** Schematic diagram of the LadderScan apparatus (upper) and perturbation assays (lower). On day 1–4, the mouse was trained to run on the walkway between two chambers. Starting from day 5 to day 8, one lower-rung (red color) was randomly elevated before the mouse reached it, with a tone cue. **(B)** Total time spent running on the rung for each trial across training days. Both the saline-treated control and the VPA-treated group showed a similar and decreasing time during the habituation phase. Starting on perturbation day 5, VPA-treated mice presented an elevated total time (*F*_(1,1496)_ = 18.88, *P* < 0.0001; Bonferroni *post hoc* comparison: *P* < 0.05 from day 6 to day 8). **(C)** Step regularity index, which is defined as the number of consecutive normal steps in each trial during a 4-day habituation followed by a 4-day perturbation. Saline-treated control mice showed gradually improved regularity, whilst VPA-treated mice showed no prominent learning effects [two-way analysis of variance (ANOVA) with respect to group effect: *F*_(1,1496)_ = 77.92, *P* < 0.0001; Bonferroni *post hoc* comparison: *P* < 0.05 from day 3 to day 8]. **(D)** Duration of each regular step (in ms) of mice on rungs across 8-day test sessions. Similar to those for the total duration, the step duration of VPA-treated mice was significantly lower after introducing perturbation (two-way ANOVA with respect to group effect: *F*_(1,1496)_ = 9.392, *P* = 0.0022; Bonferroni *post hoc* comparison: *P* < 0.05 from day 5 to day 8). **(E)** The percentage of distribution of responses toward perturbated rungs on each daily session, including jump over, step on, blockade and no perturbation. ^*^P < 0.05; *N* = 125 trials from five mice in saline and VPA group.

To further elaborate motor learning function, we employed the LadderScan apparatus (Clever System Inc., Reston, VA, USA; Figure 1A). The design of this learning paradigm was modified from a previous report (Vinueza Veloz et al., 2015). The whole apparatus consists of one runway and two side-chambers, which are equipped with LED lights and fans. The runway includes 37 rungs (metal rods, 2 mm diameter, in two lines) with a 15 mm interval. On one side, rungs with an odd number (1, 3, 5, …, 37) were at a high level, whilst even numbered rungs (2, 4, 6, …, 36) were located at a lower level. There were alternative patterns (odd number at low, even number at high level) on the other side. The vertical distance between high and low rungs was 6 mm in height, and mice typically walked on the high rungs only. During the middle segment (rung 7–31), each lower rung was elevated by 18 mm to create one extra obstacle with a 12 mm height. All rungs were equipped with pressure sensors to time each gait. One single trial was initiated after placing the mouse into the chamber on either side. After a 9–12 s delay, light and air puffs were sequentially applied in the chamber to create negative stimuli, which forced the mouse to leave the chamber. The trial was terminated when the mouse successfully walked across the runway to reach the chamber on the other side. The whole behavioral paradigm started with a 4-day training run (25 daily trials for each mouse) to ensure the mouse ran smoothly on the runway. Starting on day 5 to day 8 (25 trials per day), one extra perturbation was introduced and consisted of one randomly “pop-up” rung ahead of the mouse. The elevation of rung occurred at any lower rung in the middle segment of the runway and was induced before a mouse approached, to avoid any spatial specific memory. The rung elevation was preceded by a tone cue (80 dB, 1,000 Hz, 250 ms duration, 250 ms before perturbation). The perturbation event occurred when the mouse performed at least three consecutive “regular steps,” which were defined as having a step length with two or four rungs. All the gait parameters were recorded for each trial and were analyzed.

### EdU Incorporation Assay

EdU assay was employed to describe the migration of the GC precursors. Following previously documented methods (Wang et al., 2017), EdU (50 mg/kg, from Click-iT EdU Alexa Fluor 594 Imaging Kit, Invitrogen, Waltham, MA, USA) was injected into the peritoneal cavity of P7 mice. The whole brain was then harvested at 2 h or 72 h later and was prepared in 8 μm cryo-sections. The staining procedure followed manual instruction of the Click-iT EdU Alexa Fluor 594 Imaging Kit. A fluorescent microscope (Zeiss, Germany) was used to capture images. The number of EdU-positive cells in the EGL and internal granular layer (IGL) was counted by the ImageJ 1.48 (NIH, Bethesda, MD, USA).

### TUNEL Assay

We used a terminal deoxynucleotidyl transferase (TdT) dUTP nick-end labeling (TUNEL) assay to measure the apoptosis of the cerebellar neurons and precursor cells. The assay was performed using an *in situ* Cell Death Detection Kit (Roche, Swiss) following its manual instructions. In brief, cryo-sections (8 μm thickness) were permeabilized using a 0.1 M sodium citrate buffer (with 0.1% Triton X-100), followed by PBS washing. A freshly prepared TUNEL reaction buffer (50 μL per slide) was added at 37°C incubation for 1 h. After PBS rinsing, the DAPI (Roche, Switzerland) was used for nuclei staining. Fluorescent images were captured as previously described.

### Golgi Staining and Sholl Analysis

Golgi staining was performed using the Rapid Golgi Stain Kit (FD NeuroTech Inc., Ellicott City, MD, USA) according to the manual’s instructions. The Purkinje cells at the apical region of the cerebellar lobule were imaged using a bright field microscope (Zeiss, Germany). NeuroLucida software (MBF Bioscience, Williston, VT, USA) was used to plot the soma and dendrites of the Purkinje cells under a manually assisted mode. The Sholl analysis was adopted using previous methods (Wang et al., 2017). Briefly, a series of concentric circles (10 μm interval) were plotted around the soma. The number of intersections of dendrites against each circle, and the total dendrite lengths within each 10 μm segment, were quantified using NeuroLucida software.

### Immunofluorescence Staining

Mice were deeply anesthetized by isoflurane and were perfused with 4% paraformaldehyde (PFA). Whole brain tissues were extracted and were dehydrated in a 30% sucrose solution at 4°C overnight. Brain slices (30 μm thickness) were prepared using a cryostat (Leica, Germany). Slices were rinsed in 0.01 M PBS and blocked in 3% FBS (with 0.1% Triton X-100) for 1 h. Primary antibody against calbindin D-28K (Abcam, Cambridge, MA, USA) was added for 48 h incubation at 4°C. Donkey anti-rabbit Alexa 488 secondary antibody (Life Technology, Camarillo, CA, USA) was added at room temperature for 2 h incubation. After PBS rinsing, DAPI was added for nuclear staining. Fluorescent images were taken for cell enumeration by the ImageJ (NIH).

### Western Blotting

The cerebellar tissues were homogenized by a RIPA buffer. Equal amounts of proteins were separated by an SDS-PAGE and were transferred to a PVDF membrane (GE Healthcare, Chicago, IL, USA). The membrane was blocked by a 5% bovine serum albumin (BSA) for 2 h and was incubated in a primary antibody against BDNF, p-TrkB (p-tyrosine kinase B), t-TrkB and β-actin (Cell Signaling Technology, Danvers, MA, USA) overnight at 4°C. The membrane was then incubated in horseradish peroxidase (HRP) conjugated anti-IgG antibody (Cell Signaling) for 2 h at room temperature. Protein bands were visualized by an ECL chemiluminescence substrate. A protein imaging system (Bio-Rad, Hercules, CA, USA) was used to capture images, and their integrated density was measured by the ImageJ (NIH).

### Electrophysiological Recordings

Mice (P18–P21) were anesthetized with isoflurane and decapitated. Sagittal slices (300 μm) were prepared in ice-cold “cutting solution” containing (in mM): 240 sucrose, 2.5 KCl, 10 D-Glucose, 26 NaHCO_3_, 1.25 Na_2_HPO_4_, 2 MgCl_2_ and 1 CaCl_2_, using a Leica VT1200S Vibratome. The slices were incubated in a submerged chamber containing an equal volume of cutting solution and recording solution, at 32 ± 1°C for 30 min and subsequently at room temperature. The recording solution contained (in mM): 126 NaCl, 26 NaHCO_3_, 10 D-glucose, 1.25 NaH_2_PO_4_, 2.5 KCl, 2 CaCl_2_ and 2 MgCl_2_. All solutions were bubbled with 95% O_2_ and 5% CO_2_ and maintained at 7.4 pH. For the whole cell recordings, slices were perfused with the recording solution at room temperature. The Purkinje cells were visualized using an infrared differential interference contrast with a Nikon Eclipse FN-1 microscope with a 40× water-immersion objective. Recordings were performed using the MultiClamp 700B (Molecular Devices, San Jose, CA, USA). The recording electrodes (4–8 MΩ) were filled with an intracellular solution containing (in mM): 126 K-gluconate, 4 KCl, 10 HEPES, 4 Mg-ATP, 0.3 Na-GTP, 10 PO-creatinine (pH 7.25 with an osmolarity of 295 ± 5). Data were low-pass filtered at 3 kHz and digitized with the DigiData 1550B using a pClamp 10 at 10 kHz sampling frequency. Miniature excitatory postsynaptic currents (mEPSCs, in present of 1 μM tetrodotoxin) were recorded as inward currents at a holding potential of −70 mV and miniature inhibitory postsynaptic currents (mIPSCs) were recorded as outward currents at a holding potential of 0 mV. Both mEPSCs and mIPSCs were measured using automated event detection in the Clampfit software (Molecular Devices, San Jose, CA, USA) using 5.5 as the match threshold, followed by a manual inspection to exclude any artifacts. All events were extracted from the records with 2 min durations. For plotting the cumulative distribution curve, 40 events were extracted from the recording series of one neuron. The averaged amplitudes and frequencies were calculated from all events in each neuron.

### Statistical Analysis

All data were presented as mean ± standard error of means (SEM), unless otherwise specified. Each group of data were first tested for normality using the KS test. Data that did not fit the Gaussian distribution, were compared by a non-parametric Mann-Whitney *U* test. For data with a normal distribution, a 2-sample student *t*-test was used for comparison between the two groups. A one-way or two-way analysis of variance (ANOVA), with repeated measures, was used for comparison among the multiple groups, depending on the number of independent variables. The Tukey or Bonferroni *post hoc* comparison was used to compare means between two specific groups after one-way or two-way ANOVA, respectively.

## Results

### Impaired Motor Learning Function by Prenatal VPA Exposure

The cerebellum is closely related with the acquisition of complex motor skills. We thus employed different motor learning paradigms to study the cerebellar associated motor functions on adult (P56) mice. The classical Rotarod assay showed that compared to the saline-treated controls, the VPA-treated group generally performed worse, especially at later training sessions (Supplementary Figure 1A). This impaired motor skill seems to not be caused by a general motor deficit, as the VPA-treated mice showed a similar movement distance in an open-field assay (Supplementary Figure 1B). However, as no significant difference was found in the learning ability on the Rota-rod (two-way ANOVA with repeated measures, interaction effect, *F*_(5,120)_ = 0.9056, *P* = 0.4799, Supplementary Figure 1A), more sensitive motor learning paradigms were expected. We thus introduced a tone-conditioned complex motor learning paradigm named the LadderScan (Figure 1A, see “Materials and Methods” section for detailed information) which was modified from a previous report (Vinueza Veloz et al., 2015). The whole test paradigm consisted of a 4-day habituation session followed by a second 4-day perturbation session. In general, VPA-treated mice showed a similar time duration completing a single trial compared to the saline-treated control group during the habituation stage (Figure 1B), and both groups showed decreased durations, indicating a normal motor function in the VPA-treated mice. After introducing the perturbation, however, the VPA-treated group showed a sharp increase in duration on the walkway, which was maintained at relatively low levels in the control group (two-way ANOVA, treatment × training day effect: *F*_(7,1496)_ = 2.54, *P* = 0.0134; Bonferroni *post hoc* comparison between VPA- and saline-treated group: *P* = 0.0137, 0.0479 and 0.0371 from day 6 to day 8; Figure 1B). Therefore, the VPA-treated mice seem to have deficits in the acquisition of this complex motor task with perturbation. When checking the step regularity index, which was defined as the number of consecutively regular steps in a single trial, the VPA-treated mice did not improve significantly with repeated training as compared to the control group (two-way ANOVA, treatment × training day effect: *F*_(7,1496)_ = 2.232, *P* = 0.0294; Bonferroni *post hoc* comparison: *P* = 0.0018, 0.0024, 0.0001, 0.0253, 0.0173 and 0.0005 from day 3 to day 8; Figure 1C). Similar results were obtained when checking the average duration of each regular step, which was continuously decreased in both the VPA-treated mice and the control group, with repeated training, but was re-elevated after perturbation introduction only in the VPA-treated group (two-way ANOVA, treatment × training day effect: *F*_(7,1496)_ = 2.524, *P* = 0.0139; Bonferroni *post hoc* comparison: *P* = 0.0423, 0.0374, 0.0109 and 0.0035 from day 5 to day 8; Figure 1D). We also analyzed the response of animals toward perturbation and found that the VPA-treated mice had a lower rate of successfully crossing the perturbation (“jump over”) compared to the control group (4.8%–10.4% vs. 9.6%–16.8%, *t*_(6)_ = 3.232, *P* = 0.0179; Figure 1E). Moreover, the VPA-treated mice presented a higher rate of “step on” on the elevated rung (31.2%–40.8% of all sessions at day 5 to day 8) compared to the saline-treated control group (6.4%–24.0%, *t*_(6)_ = 5.024, *P* = 0.0024; Figure 1E). In summary, the LadderScan paradigm suggested that the VPA-treated mice developed motor incoordination when faced with unexpected perturbation, while they maintained normal motor function during relatively simple motor training. Such impairment in complex motor learning did not improve significantly with repeated training. These results supported the impaired motor learning after prenatal VPA exposure.

### Retardation of Motor Function Development at Early Postnatal Stage in VPA-Treated Mice

**Figure 2:**
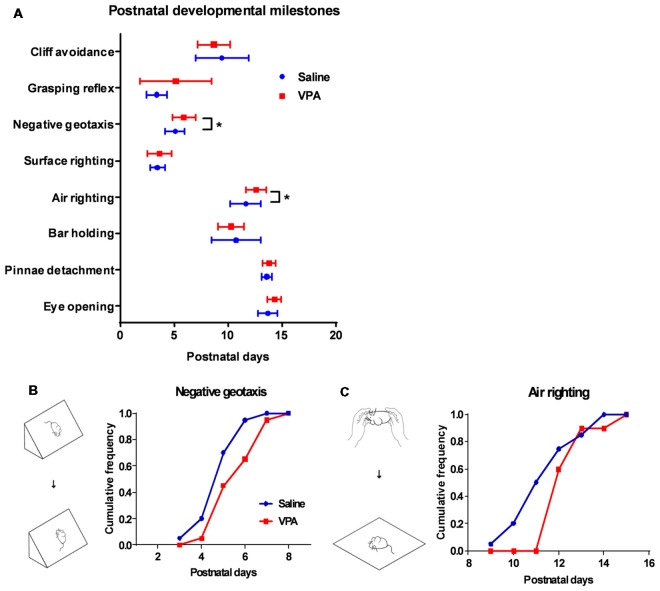
Retardation of motor reflex onset in juvenile VPA-treated mice. **(A)** Days of onset for major developmental milestones in juvenile mice between the saline-treated control and the VPA-treated groups. In negative geotaxis (Mann Whitney test, *U* = 122.5, *P* = 0.0275) and air righting motor reflex (*U* = 114, *P* = 0.0139), VPA-treated mice showed delayed days of onset. **(B)** Schematic illustration for negative geotaxis (left). The juvenile mouse was placed on an inclined plane with the head facing down, and a positive reflex was defined when the mouse recovered to the head-up position. Right panel, cumulative frequency of mice having a positive reflex across the postnatal days. **(C)** Left panel, schematic illustration for air righting reflex, which is defined as turning around its body when the mouse was released in a supine position. Right panel, cumulative frequency of days of onset for air righting reflex. ^*^P < 0.05; *N* = 20 juveniles from the saline-treated and VPA-treated group.

Having observed the motor learning deficit in adult VPA-treated mice, we next explored if such impairment occurred at an early age. A series of developmental “milestones” was thus employed to describe both body development and motor reflex patterns during the first three postnatal weeks (Zhang et al., 2014). In examining two markers for the body development: pinnae detachment and eye opening, we found similar days of onset between the VPA- and saline-treated mice (Figure 2A), indicating unaltered general development. We further investigated the onset of several sensorimotor reflexes in juvenile mice including cliff avoidance, grasping, negative geotaxis, surface righting, air righting and bar holding. Among those markers, we found that prenatal exposure of VPA, led to the retardation of the negative geotaxis reflex, which is defined as the ability to turn the body around from a head-down position on an inclined plane. The VPA-treated mice also presented a late onset of the air righting reflex, in which the mouse can adjust its body to a prone position when landing on soft beddings, after releasing from a height in a supine posture. Statistical analysis showed the late onset of both reflexes in the VPA-treated group (Negative geotaxis: 5.9 ± 0.24 vs. 5.1 ± 0.20 days, Mann Whitney test, *U* = 122.5, *P* = 0.0275; Air-righting reflex: 12.6 ± 0.21 vs. 11.6 ± 0.32 days, *U* = 114, *P* = 0.0139; *n* = 20 in each group; Figures 2B,C). As previous reports have illustrated the impairment of the negative geotaxis (Holmes et al., 2006) and the air righting reflex (Wolf et al., 1996) in cerebellar developmental disorders, it is thus reasonable to speculate that prenatal VPA exposure may cause early postnatal developmental disorders of the cerebellum.

### Aberrant Cerebellar Cortical Formation in a VPA-Treated Mouse

**Figure 3:**
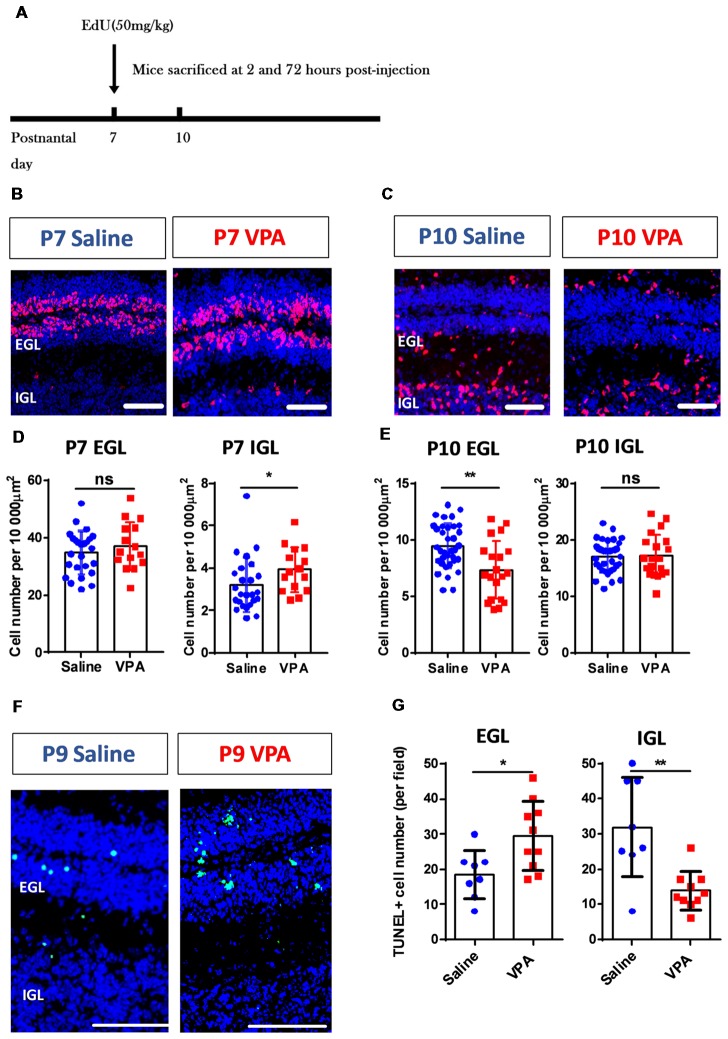
Aberrant cerebellar cortical development in VPA-treated mice during the early postnatal phase.** (A)** Timelines for the EdU labeling and sampling schedule. **(B,C)** Representative images for the EdU+ cells (red) with DAPI (blue) staining in sagittal sections of the cerebellum at P7 **(B)** and P10 **(C)**. **(D,E)** Quantitative analysis for the density of the EdU+ cells (per 10,000 μm^2^) in the external granular layer (EGL) and internal granular layer (IGL) on P7 **(D)** and P10 **(E)**. VPA-treated mice showed more EdU+ cells in P7 IGL (Mann Whitney test, *U* = 104, *P* = 0.0191) and fewer cells in P10 EGL (2-sample student *t*-test, *t*_(53)_ = 3.416, *P* = 0.0012), suggesting a pre-onset of the granular cell (GC) precursors migration. *N* = 25 slices from six mice in the saline-treated group, and 15 slices from five mice in the VPA-treated group. **(F)** Representative images for terminal deoxynucleotidyl transferase (TdT) dUTP nick-end labeling (TUNEL) staining to label apoptotic cells (green) at P9. **(G)** Quantitative analysis of apoptotic cells. VPA-treated mice showed more TUNEL+ cells in the EGL (*t*_(16)_ = 2.673 *P* = 0.0167) but less apoptosis in the IGL (*t*_(16)_ = 3.75). *N* = 8 slices from three mice in the saline-treated group, and 10 slices from four mice in the VPA-treated group. ns, no significant difference; ^*^P < 0.05; ^**^P < 0.01. Scale bar, 100 μm in **(B,C,F)**.

The abovementioned motor deficits and retardation of motor reflexes in the VPA-treated mice, indicate altered cerebellar development patterns. As the most abundant neuron type, the GC forms the major excitatory input to the Purkinje cells via a parallel fiber, in addition to a climbing fiber originating from the inferior olive nuclei. During early postnatal stage, GC precursors in the EGL undergo active proliferation followed by an inwardly radial migration to the IGL where they differentiate into mature GCs (Butts et al., 2014; Marzban et al., 2015). Using the EdU incorporation assay, we tracked the migration of newly generated GC precursors between P7 and P10 (Figure 3A). The VPA-treated mouse juveniles displayed significantly more EdU+ cells in the IGL at P7 (Mann Whitney test, *U* = 104, *P* = 0.0191; Figures 3B,D) but less cells in the EGL at P10 compared to the saline-treated control group (*t*_(53)_ = 3.416, *P* = 0.0012; Figures 3C,E). No difference was found in the P7 EGL or P10 IGL (*P* = 0.3545 and 0.8849). Since the GC precursors migrate from the EGL to the IGL, it indicates that VPA-treated mice presented premature inward migration. Newly formed cells also undergo programmed cell death if not undergoing migration or maturation. We further used a TUNEL assay and found that the VPA-treated mice showed elevated apoptosis at P9 in the EGL, but fewer deaths in the IGL (EGL: *t*_(16)_ = 2.673, *P* = 0.0167; IGL: *t*_(16)_ = 3.75, *P* = 0.0017; Figures 3F,G). Such accelerated apoptosis persisted until P15 in the EGL but not for the IGL (Supplementary Figure S2). Combining these results, prenatal VPA exposure leads to premature GC precursor migration in the cerebellar cortex during the second postnatal week, in association with higher apoptosis.

### Purkinje Cell Deformation After Prenatal VPA Exposure

**Figure 4:**
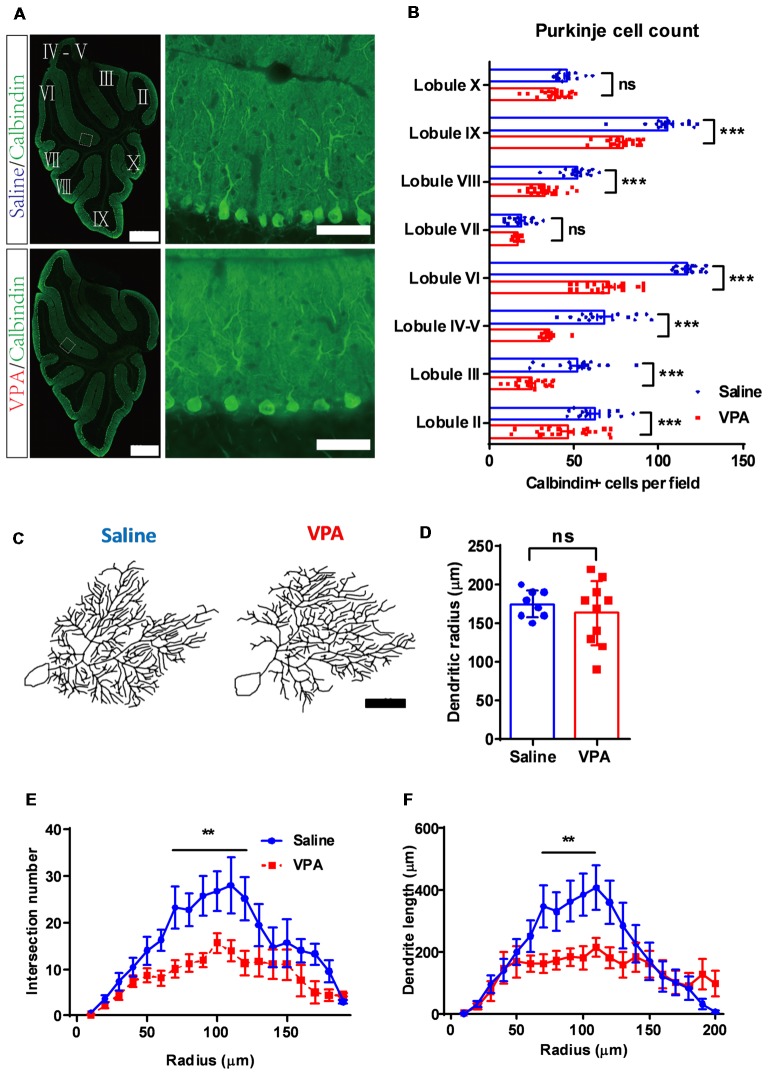
Purkinje cell de-population and impaired dendritic arborization in VPA-treated mice.** (A)** Representative images for the whole cerebellum in a sagittal view (left panels) and high magnification images showing the cerebellar cortex (right panels) from the saline-treated control (upper) and the VPA-treated group (lower). Cerebellar lobules were labeled with Roman numbers. **(B)** Quantitative analysis for the Purkinje cell number in each lobule. Statistical analysis showed lower Purkinje cell numbers in most lobules examined (two-way ANOVA with respect to group effect: *F*_(1,265)_ = 248.5, *P* < 0.0001; Bonferroni *post hoc* comparison: *P* < 0.001 for all lobules but lobule VII and lobule X). *N* = 16 slices from four mice in the saline-treated group, and 22 slices from five mice in the VPA-treated group. **(C)** Re-plotting of the Purkinje cell dendritic trees at P30. **(D)** No significant difference of dendritic tree radius between the saline-treated control and VPA-treated group (2-sample student *t*-test, *t*_(16)_ = 0.7583, *P* = 0.4593). **(E,F)** Sholl analysis showed that VPA-treated mice had a decreased complexity of the dendritic trees in the middle segment, as suggested by fewer intersection numbers (two-way ANOVA with respect to group effect; intersection number: *F*_(1,235)_ = 56.64, *P* < 0.0001; Bonferroni *post hoc* comparison: *P* < 0.05 from 70 μm to 120 μm segment; **E**) and shorter dendrite length (*F*_(1,235)_ = 17.85, *P* < 0.0001; Bonferroni *post hoc* comparison: *P* < 0.05 from 70 μm to 100 μm segment; **F**). *N* = 12 cells from three mice in the saline-treated group, and 16 cells from four mice in the VPA-treated group. ns, no significant difference; ^**^P < 0.01; ^***^P < 0.001. Scale bar, 500 μm (low magnification) and 50 μm (high magnification) in **(A)**, and 50 μm in **(C)**.

Cerebellar development is one well-orchestrated process involving neurogenesis, dendritic arborization and synaptogenesis. After demonstrating premature migration and excessed apoptosis of GC precursors, we next investigated the Purkinje cells. In adult mice, we found a significantly decreased number of Purkinje neurons in most of the lobules (two-way ANOVA, group effect: *F*_(1,265)_ = 248.5, *P* < 0.0001; Bonferroni *post hoc* comparison: *P* < 0.001 for all lobules but lobule VII and lobule X, Figures 4A,B). On average, the Purkinje cell number was decreased by 15.3%–52.3% across the lobules in the VPA-treated mice, suggesting an impaired Purkinje cell population. Due to normal cerebellar size and lobular formation of the cerebellum (Figure 4A), VPA exposure decreased the Purkinje cell density. Moreover, we examined the dendritic formation of the Purkinje cells and found that at P9, the VPA-treated mice showed a lower dendritic field radius (Supplementary Figure S3). A Sholl analysis indicated altered dendritic arborization patterns at this stage, as indicated by the higher complexity in the proximal dendritic segment but fewer branches at the distal site (Supplementary Figure S3). At the adult stage (P30), the Purkinje cells in the VPA-treated group presented a similar dendritic field radius compared to the control group (*t*_(16)_ = 0.7583, *P* = 0.4593; Figures 4C,D). However, the dendritic arborization was remarkably impaired, especially at the middle-to-distal segment (70–120 μm from the soma) of the dendritic tree, as suggested by fewer intersections or shorter dendrite lengths (intersection number: two-way ANOVA, group effect; *F*_(1,235)_ = 56.64, *P* < 0.0001; Bonferroni *post hoc* comparison: *P* < 0.05 from 70 μm to 120 μm segment; Figures 4C,E. Dendrite length: *F*_(1,235)_ = 17.85, *P* < 0.0001; Bonferroni *post hoc* comparison: *P* < 0.05 from 70 μm to 100 μm segment; Figures 4C,F). The data therefore suggested that prenatal VPA exposure ablated the normal population and dendritic formation of the Purkinje neurons, in a progressive manner, from the juvenile until the adult stage.

### Impaired Synaptic Transmission of Purkinje Neurons in the VPA Model

**Figure 5:**
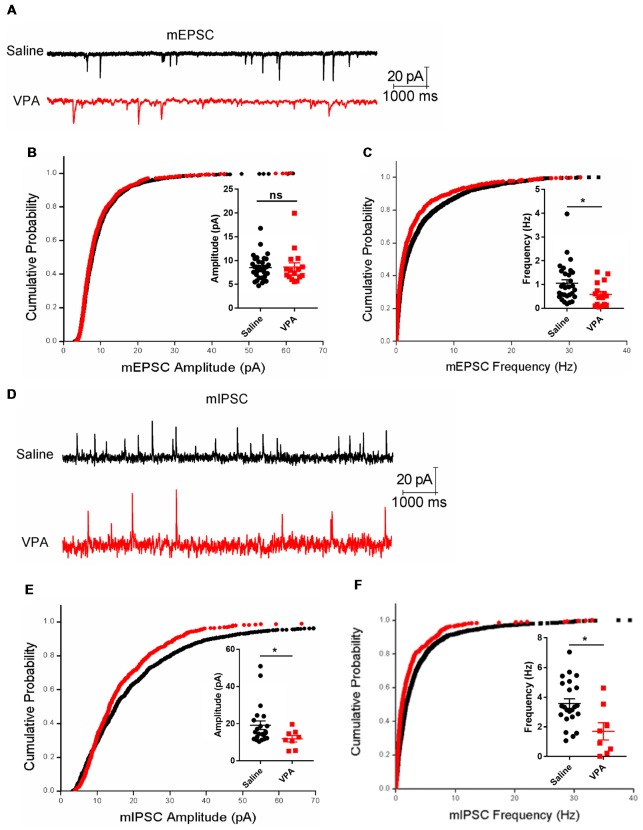
Impaired synaptic transmission in the cerebellar cortex.** (A)** Representative traces from the whole-cell voltage-clamp recording of the miniature excitatory postsynaptic current (mEPSC) in the Purkinje neurons from a saline-treated (upper) and VPA-treated mouse (lower). **(B)** Cumulative distribution of mEPSC amplitude and plotted averaged amplitudes (insert figure) from each recorded neuron. **(C)** Same as **(B)** but for the instantaneous frequency of mEPSC. *N* = 33 neurons from five mice in the saline-treated group, and 16 neurons from four mice in the VPA-treated group. **(D)** Extracted traces for the miniature inhibitory postsynaptic current (mIPSC) recording. **(E)** Cumulative distribution of mIPSC amplitude and plotted averaged values. **(F)** Same as **(E)** but for mIPSC instantaneous frequency. *N* = 22 neurons from five mice in the saline-treated group, and eight neurons from four mice in the VPA-treated group. In all cumulative curves, 40 events were extracted from each neuron for statistical analysis. ns, no significant difference; ^*^P < 0.05 using Mann-Whitney nonparametric comparison.

The Purkinje neurons receive excitatory synaptic inputs from the GCs via parallel fibers and from climbing fibers originating from the inferior olive nuclei, as well as inhibitory inputs from the interneurons in the molecular layers. Therefore, the impaired dendritic arborization of the Purkinje cell in the VPA-treated mouse model may lead to an aberrant synaptic transmission. To test this hypothesis, we prepared acute cerebellar slices from both VPA- and saline-treated mice and recorded the excitatory and inhibitory transmission on the Purkinje cells. When examining the mEPSC, the VPA-treated group had unchanged amplitudes (Mann Whitney test, *U* = 237, *P* = 0.5759; Figures 5A,B) but remarkably decreased frequencies compared to the control group (0.58 ± 0.12 Hz vs. 1.06 ± 0.15 Hz, *U* = 136, *P* = 0.0155; Figure 5C). This evidence strongly supports the impaired excitatory transmission in the cerebellar cortex. We also examined the mIPSC, which is evoked by the interneurons including the basket cells and stellate cells in the molecular layer. VPA-treated mice also showed decreased mIPSC amplitudes (11.88 ± 1.73 pA vs. 19.17 ± 2.28 pA, *U* = 45, *P* = 0.0446; Figures 5D,E) and suppressed frequencies (1.74 ± 0.53 Hz vs. 3.57 ± 0.32 Hz, *U* = 54, *P* = 0.0155; Figure 5F). In sum, prenatal VPA exposure led to suppressed excitatory and inhibitory transmission, which is consistent with the morphological alternations and motor learning deficits mentioned above.

## Discussion

The current study demonstrates the impaired postnatal development of the cerebellar cortex after prenatal VPA exposure, in association with motor deficits during both the juvenile and adult stages. Although having no prominent effect on the gross volume or foliation of the cerebellum, single prenatal VPA injection does lead to a premature GC precursor migration and an excess apoptosis, as well as a lower Purkinje cell density or an impaired dendritic arborization. These structural defects are associated with impaired synaptic transmission in the Purkinje cells. In order to validate ASD-like behaviors in our VPA model, we performed a 3-chamber assay, in which male VPA-treated mice showed an impaired sociability or social novelty (Supplementary Figure S4), as consistent with previous reports (Roullet et al., 2010). Furthermore, the retardation of motor reflexes occurred in our VPA-induced mouse ASD model, agreeing with clinical observations showing motor deficits in ASD children before the onset of social or verbal disorders (Lloyd et al., 2013). Our animal data thus supports the examination of early motor disorder as a marker in ASD screening (Brisson et al., 2012; Zwaigenbaum et al., 2013; Sacrey et al., 2015).

Deficits of motor coordination and motor learning have been reported after postnatal VPA exposure (Pragnya et al., 2014). Here, we showed similar phenotypes using prenatal VPA treatment, as suggested by both Rota-rod and LadderScan assays. Such consistent results from our prenatal exposure model as well as the postnatal models of other groups (Kim et al., 2013) suggest that VPA affects both the prenatal and postnatal development of the cerebellum. The tone-conditioned motor learning of a perturbation during walking paradigms, has been established as tightly associated with cerebellar functions (Van Der Giessen et al., 2008). Using similar behavioral approaches, a recent study reported neonatal hypoxia-induced aberrant cerebellar motor learning, as suggested by a higher percentage of irregular steps and longer step durations with the perturbation (Sathyanesan et al., 2018). Both of those phenotypes have been replicated in our VPA model and thus supports the cerebellar deformation and dysfunction. The motor deficits in hypoxic mice were further attributed to a decreased frequency of simple spike firings of the Purkinje cells (Sathyanesan et al., 2018). We consistently showed decreased mEPSC frequencies as well as lower mIPSC frequencies or amplitudes in VPA-treated mice, indicating impaired presynaptic inputs from the parallel fibers or climbing fibers, and from the inhibitory interneurons. The impaired synaptic transmissions may be caused by deficits of dendritic arborization of the Purkinje cells. Apart from those structural deformations, neurotransmitter imbalance may also be involved in ASD-induced cerebellar pathology. A previous study reported a decreased expression of 65 and 67 kDa enzymes of l-glutamic acid decarboxylase (GAD65/67) in a rat cerebellum after prenatal VPA exposure (Olexová et al., 2016). Hippocampal tissues in VPA-treated rats also have decreased glutamine synthesis and impaired glutamate uptake (Bristot Silvestrin et al., 2013). These two separate lines of evidence also supported our observations showing defects of presynaptic excitatory and inhibitory transmissions in the cerebellar cortex of VPA-treated mice. Our anatomical, electrophysiological and behavioral assays therefore provide consistent results showing disrupted cerebellar structures and functions after prenatal VPA exposure.

As a neurodevelopmental disorder, ASD influences neural circuit formation across various brain regions (Mohammad-Rezazadeh et al., 2016), among which the cerebellum has repeatedly been identified (Wang et al., 2014). Such evidence, however, mostly come from transgenic mouse models (Cupolillo et al., 2016; Peter et al., 2016) or ASD patients (Hampson and Blatt, 2015). For example, decreased gray matter volumes (D’Mello et al., 2015) and impaired microstructural connectivity (Hanaie et al., 2013) can be found in the cerebellum of ASD individuals. In mice carrying *Shank2* mutation in the Purkinje cells, potentiated postsynaptic inhibitory transmission as well as impaired long-term potentiation (LTP) have been associated with motor learning and social deficits (Peter et al., 2016). Nevertheless, our understanding of the effects of ASD-related environmental factors on the cerebellum is far from complete. Prenatal VPA exposure is one commonly used rodent ASD model for mechanistic and therapeutic investigations (Nicolini and Fahnestock, 2018). Previous studies have reported elevated apoptosis of GC precursors (Yochum et al., 2008, 2010) and impaired motor functions (Pragnya et al., 2014) after postnatal VPA exposure. Our study, on the other hand, provides evidence showing interruptions of postnatal cerebellar development through prenatal VPA administration, including the premature migration and excess apoptosis of the GC precursors, and the deformation of the Purkinje cells. GC precursors are highly vulnerable to environmental stimuli. Ethanol exposure, for example, elevates GC apoptosis at an early postnatal stage (Oliveira et al., 2014) and causes motor dysfunctions (Valenzuela et al., 2010). Our VPA model also displayed higher GC apoptosis, as well as pre-onset of GC migration from the EGL to the IGL. The premature GCs migration was previously found to be associated with elevated cell apoptosis (Wang et al., 2017), probably due to an unfavorable micro-niche for the immature neuron precursors. More importantly, matured GCs form one of the two major excitatory inputs on the Purkinje cells. Therefore, GC survival and maturation is crucial for dendrite formation of the Purkinje cells in an activity-dependent manner (as reviewed in Tanaka, 2009). We thus expect that aberrant GC formation and maturation in VPA-treated mice can contribute to Purkinje cell deformation as suggested by morphological studies. Taken together, a decreased Purkinje cell number and dendrite arborization, as well as previously uncharacterized synaptic dysfunction, can help explain the impaired motor learning in VPA-treated mice.

The molecular mechanisms explaining VPA toxicity on the cerebellum are currently still inconclusive. Owing to its functional roles as one histone deacetylase (HDAC) inhibitor, VPA can mediate the expression of various target genes. Therefore, a wide spectrum of pathological phenotypes may co-exist after VPA injection. Some opinions state that a central inflammatory response is involved, as suggested by microglial activation (Suzuki et al., 2013) and elevated proinflammatory cytokines (Lucchina and Depino, 2014). VPA can also alter the endocannabinoid system (Kerr et al., 2013) or the androgen receptor (Perez-Pouchoulen et al., 2016) in the cerebellum. The neurotrophic factor may form an alternative explanation and we found an elevated expression of the brain derived neurotrophic factor (BDNF) in the P15 cerebellum but no change of its receptor TrkB (Supplementary Figure S5). In the VPA-treated mouse fetus (Almeida et al., 2014) and in ASD patients (Ricci et al., 2013), excess BDNF have been observed and may be related to ASD neuropathology. In addition to BDNF hypothesis, VPA also mediates the expression of the neurodevelopmental genes such as *Neuroligin1*, *Shank2* and *Shank3* to delay neuronal maturation (Kawanai et al., 2016). Further studies should thus be performed to investigate the major molecular pathways for postnatal cerebellar development such as *sonic hedgehog (Shh)*, to elaborate the developmental effects of VPA. In addition, the teratogenic effects of VPA on the cerebellum may not be limited to the cortical region, as embryonic VPA exposure disrupts normal cell population and nuclei morphology of the deep cerebellar nuclei (DCN; Mowery et al., 2015). More investigations on the effect of VPA on cerebellar circuitry formation could thus help to further elaborate the role of the cerebellum in ASD pathology.

In summary, the current study provides a series of systematic observations for morphological, electrophysiological and functional defects of the cerebellar cortex after prenatal VPA exposure. VPA effectively alters the migration pattern and survival of GC precursors, and leads to a lower Purkinje cell density and impaired dendritic arborizations. Such anatomical deficits are associated with impaired synaptic transmission, which probably lead to motor deficits from the early postnatal stages until the adult stages. This study illustrates the teratogenic effects of VPA on the cerebellum and helps to better characterize the cerebellar neuropathology of ASD. In a clinical perspective, the previously uncharacterized retardations of motor reflexes in VPA-treated juvenile mice supports the exploration of using motor examination in early ASD screening.

## Author Contributions

RW and JT designed and performed all the experiments. JG, YZ and QH performed the morphological works. JY designed and performed the electrophysiological studies. LZ and K-FS wrote the manuscript and supervised all the experiments. All authors revised and approved the manuscript.

## Conflict of Interest Statement

The authors declare that the research was conducted in the absence of any commercial or financial relationships that could be construed as a potential conflict of interest.

## References

[B1] Almeida L. E. Roby C. D. Krueger B. K. (2014). Increased BDNF expression in fetal brain in the valproic acid model of autism. Mol. Cell. Neurosci. 59, 57–62. 10.1016/j.mcn.2014.01.007 24480134 PMC4008664

[B2] Brisson J. Warreyn P. Serres J. Foussier S. Adrien-Louis J. (2012). Motor anticipation failure in infants with autism: a retrospective analysis of feeding situations. Autism 16, 420–429. 10.1177/1362361311423385 22250193

[B36] Bristot Silvestrin R. Bambini-Junior V. Galland F. Daniele Bobermim L. Quincozes-Santos A. Torres Abib R.. (2013). Animal model of autism induced by prenatal exposure to valproate: altered glutamate metabolism in the hippocampus. Brain Res. 1495, 52–60. 10.1016/j.brainres.2012.11.048 23219577

[B3] Butts T. Green M. J. Wingate R. J. (2014). Development of the cerebellum: simple steps to make a ‘little brain’. Development 141, 4031–4041. 10.1242/dev.106559 25336734

[B4] Codagnone M. G. Podestá M. F. Uccelli N. A. Reinés A. (2015). Differential local connectivity and neuroinflammation profiles in the medial prefrontal cortex and hippocampus in the valproic acid rat model of autism. Dev. Neurosci. 37, 215–231. 10.1159/000375489 25895486

[B5] Cupolillo D. Hoxha E. Faralli A. De Luca A. Rossi F. Tempia F.. (2016). Autistic-Like traits and cerebellar dysfunction in Purkinje cell PTEN knock-out mice. Neuropsychopharmacology 41, 1457–1466. 10.1038/npp.2015.339 26538449 PMC4832032

[B6] D’Mello A. M. Crocetti D. Mostofsky S. H. Stoodley C. J. (2015). Cerebellar gray matter and lobular volumes correlate with core autism symptoms. Neuroimage Clin. 7, 631–639. 10.1016/j.nicl.2015.02.007 25844317 PMC4375648

[B7] Fatemi S. H. Aldinger K. A. Ashwood P. Bauman M. L. Blaha C. D. Blatt G. J.. (2012). Consensus paper: pathological role of the cerebellum in autism. Cerebellum 11, 777–807. 10.1007/s12311-012-0355-9 22370873 PMC3677555

[B8] Hampson D. R. Blatt G. J. (2015). Autism spectrum disorders and neuropathology of the cerebellum. Front. Neurosci. 9:420. 10.3389/fnins.2015.00420 26594141 PMC4635214

[B9] Hanaie R. Mohri I. Kagitani-Shimono K. Tachibana M. Azuma J. Matsuzaki J.. (2013). Altered microstructural connectivity of the superior cerebellar peduncle is related to motor dysfunction in children with autistic spectrum disorders. Cerebellum 12, 645–656. 10.1007/s12311-013-0475-x 23564050

[B10] Holmes M. C. Sangra M. French K. L. Whittle I. R. Paterson J. Mullins J. J.. (2006). 11β-Hydroxysteroid dehydrogenase type 2 protects the neonatal cerebellum from deleterious effects of glucocorticoids. Neuroscience 137, 865–873. 10.1016/j.neuroscience.2005.09.037 16289840 PMC6443040

[B11] Kawanai T. Ago Y. Watanabe R. Inoue A. Taruta A. Onaka Y.. (2016). Prenatal exposure to histone deacetylase inhibitors affects gene expression of autism-related molecules and delays neuronal maturation. Neurochem. Res. 41, 2574–2584. 10.1007/s11064-016-1969-y 27300699

[B12] Kerr D. M. Downey L. Conboy M. Finn D. P. Roche M. (2013). Alterations in the endocannabinoid system in the rat valproic acid model of autism. Behav. Brain Res. 249, 124–132. 10.1016/j.bbr.2013.04.043 23643692

[B13] Kim J. E. Shin M. S. Seo T. B. Ji E. S. Baek S. S. Lee S. J.. (2013). Treadmill exercise ameliorates motor disturbance through inhibition of apoptosis in the cerebellum of valproic acid-induced autistic rat pups. Mol. Med. Rep. 8, 327–334. 10.3892/mmr.2013.1518 23760019

[B14] Lloyd M. MacDonald M. Lord C. (2013). Motor skills of toddlers with autism spectrum disorders. Autism 17, 133–146. 10.1177/1362361311402230 21610184 PMC3188325

[B15] Lucchina L. Depino A. M. (2014). Altered peripheral and central inflammatory responses in a mouse model of autism. Autism Res. 7, 273–289. 10.1002/aur.1338 24124122

[B16] Mandy W. Lai M. C. (2016). Annual research review: the role of the environment in the developmental psychopathology of autism spectrum condition. J. Child Psychol. Psychiatry 57, 271–292. 10.1111/jcpp.12501 26782158

[B17] Markram K. Rinaldi T. La Mendola D. Sandi C. Markram H. (2008). Abnormal fear conditioning and amygdala processing in an animal model of autism. Neuropsychopharmacology 33, 901–912. 10.1038/sj.npp.1301453 17507914

[B18] Marzban H. Del Bigio M. R. Alizadeh J. Ghavami S. Zachariah R. M. Rastegar M. (2015). Cellular commitment in the developing cerebellum. Front. Cell. Neurosci. 8:450. 10.3389/fncel.2014.00450 25628535 PMC4290586

[B19] McPhillips M. Finlay J. Bejerot S. Hanley M. (2014). Motor deficits in children with autism spectrum disorder: a cross-syndrome study. Autism Res. 7, 664–676. 10.1002/aur.1408 25258309

[B20] Mohammad-Rezazadeh I. Frohlich J. Loo S. K. Jeste S. S. (2016). Brain connectivity in autism spectrum disorder. Curr. Opin. Neurol. 29, 137–147. 10.1097/WCO.0000000000000301 26910484 PMC4843767

[B21] Mosconi M. W. Mohanty S. Greene R. K. Cook E. H. Vaillancourt D. E. Sweeney J. A. (2015). Feedforward and feedback motor control abnormalities implicate cerebellar dysfunctions in autism spectrum disorder. J. Neurosci. 35, 2015–2025. 10.1523/JNEUROSCI.2731-14.2015 25653359 PMC4315832

[B22] Mothersill O. Knee-Zaska C. Donohoe G. (2016). Emotion and theory of mind in schizophrenia-investigating the role of the cerebellum. Cerebellum 15, 357–368. 10.1007/s12311-015-0696-2 26155761

[B23] Mowery T. M. Wilson S. M. Kostylev P. V. Dina B. Buchholz J. B. Prieto A. L.. (2015). Embryological exposure to valproic acid disrupts morphology of the deep cerebellar nuclei in a sexually dimorphic way. Int. J. Dev. Neurosci. 40, 15–23. 10.1016/j.ijdevneu.2014.10.003 25447790

[B24] Nicolini C. Fahnestock M. (2018). The valproic acid-induced rodent model of autism. Exp. Neurol. 299, 217–227. 10.1016/j.expneurol.2017.04.017 28472621

[B25] Olexová L. Štefánik P. Kršková L. (2016). Increased anxiety-like behaviour and altered GABAergic system in the amygdala and cerebellum of VPA rats—an animal model of autism. Neurosci. Lett. 629, 9–14. 10.1016/j.neulet.2016.06.035 27353514

[B26] Oliveira S. A. Chuffa L. G. Fioruci-Fontanelli B. A. Lizarte Neto F. S. Novais P. C. Tirapelli L. F.. (2014). Apoptosis of Purkinje and granular cells of the cerebellum following chronic ethanol intake. Cerebellum 13, 728–738. 10.1007/s12311-014-0591-2 25129034

[B27] Ornoy A. (2009). Valproic acid in pregnancy: how much are we endangering the embryo and fetus? Reprod. Toxicol. 28, 1–10. 10.1016/j.reprotox.2009.02.014 19490988

[B28] Perez-Pouchoulen M. Miquel M. Saft P. Brug B. Toledo R. Hernandez M. E.. (2016). Prenatal exposure to sodium valproate alters androgen receptor expression in the developing cerebellum in a region and age specific manner in male and female rats. Int. J. Dev. Neurosci. 53, 46–52. 10.1016/j.ijdevneu.2016.07.001 27423376

[B29] Peter S. Ten Brinke M. M. Stedehouder J. Reinelt C. M. Wu B. Zhou H.. (2016). Dysfunctional cerebellar Purkinje cells contribute to autism-like behaviour in Shank2-deficient mice. Nat. Commun. 7:12627. 10.1038/ncomms12627 27581745 PMC5025785

[B30] Pragnya B. Kameshwari J. S. Veeresh B. (2014). Ameliorating effect of piperine on behavioral abnormalities and oxidative markers in sodium valproate induced autism in BALB/C mice. Behav. Brain Res. 270, 86–94. 10.1016/j.bbr.2014.04.045 24803211

[B31] Ricci S. Businaro R. Ippoliti F. Lo Vasco V. R. Massoni F. Onofri E.. (2013). Altered cytokine and BDNF levels in autism spectrum disorder. Neurotox. Res. 24, 491–501. 10.1007/s12640-013-9393-4 23604965

[B32] Roullet F. I. Lai J. K. Foster J. A. (2013). *In utero* exposure to valproic acid and autism—a current review of clinical and animal studies. Neurotoxicol. Teratol. 36, 47–56. 10.1016/j.ntt.2013.01.004 23395807

[B33] Roullet F. I. Wollaston L. Decatanzaro D. Foster J. A. (2010). Behavioral and molecular changes in the mouse in response to prenatal exposure to the anti-epileptic drug valproic acid. Neuroscience 170, 514–522. 10.1016/j.neuroscience.2010.06.069 20603192

[B34] Sacrey L. A. Bennett J. A. Zwaigenbaum L. (2015). Early infant development and intervention for autism spectrum disorder. J. Child Neurol. 30, 1921–1929. 10.1177/0883073815601500 26323499

[B35] Sathyanesan A. Kundu S. Abbah J. Gallo V. (2018). Neonatal brain injury causes cerebellar learning deficits and Purkinje cell dysfunction. Nat. Commun. 9:3235. 10.1038/s41467-018-05656-w 30104642 PMC6089917

[B37] Sosa-Díaz N. Bringas M. E. Atzori M. Flores G. (2014). Prefrontal cortex, hippocampus, and basolateral amygdala plasticity in a rat model of autism spectrum. Synapse 68, 468–473. 10.1002/syn.21759 24985713

[B38] Su L. Cai Y. Xu Y. Dutt A. Shi S. Bramon E. (2014). Cerebral metabolism in major depressive disorder: a voxel-based meta-analysis of positron emission tomography studies. BMC Psychiatry 14:321. 10.1186/s12888-014-0321-9 25407081 PMC4240898

[B39] Suzuki K. Sugihara G. Ouchi Y. Nakamura K. Futatsubashi M. Takebayashi K.. (2013). Microglial activation in young adults with autism spectrum disorder. JAMA Psychiatry 70, 49–58. 10.1001/jamapsychiatry.2013.272 23404112

[B40] Tanaka M. (2009). Dendrite formation of cerebellar Purkinje cells. Neurochem. Res. 34, 2078–2088. 10.1007/s11064-009-0073-y 19821027

[B41] Valenzuela C. F. Lindquist B. Zamudio-Bulcock P. A. (2010). A review of synaptic plasticity at Purkinje neurons with a focus on ethanol-induced cerebellar dysfunction. Int. Rev. Neurobiol. 91, 339–372. 10.1016/s0074-7742(10)91011-8 20813248

[B42] Van Der Giessen R. S. Koekkoek S. K. van Dorp S. De Gruijl J. R. Cupido A. Khosrovani S.. (2008). Role of olivary electrical coupling in cerebellar motor learning. Neuron 58, 599–612. 10.1016/j.neuron.2008.03.016 18498740

[B43] Vinueza Veloz M. F. Zhou K. Bosman L. W. Potters J. W. Negrello M. Seepers R. M.. (2015). Cerebellar control of gait and interlimb coordination. Brain Struct. Funct. 220, 3513–3536. 10.1007/s00429-014-0870-1 25139623 PMC4575700

[B45] Wang S. S. Kloth A. D. Badura A. (2014). The cerebellum, sensitive periods, and autism. Neuron 83, 518–532. 10.1016/j.neuron.2014.07.016 25102558 PMC4135479

[B44] Wang L. Zhang L. Chow B. K. C. (2017). Secretin modulates the postnatal development of mouse cerebellar cortex Via PKA- and ERK-dependent pathways. Front. Cell. Neurosci. 11:382. 10.3389/fncel.2017.00382 29249942 PMC5714926

[B46] Whyatt C. Craig C. (2013). Sensory-motor problems in Autism. Front. Integr. Neurosci. 7:51. 10.3389/fnint.2013.00051 23882194 PMC3714545

[B47] Wolf L. W. LaRegina M. C. Tolbert D. L. (1996). A behavioral study of the development of hereditary cerebellar ataxia in the shaker rat mutant. Behav. Brain Res. 75, 67–81. 10.1016/0166-4328(96)00159-3 8800661

[B48] Yochum C. L. Bhattacharya P. Patti L. Mirochnitchenko O. Wagner G. C. (2010). Animal model of autism using GSTM1 knockout mice and early post-natal sodium valproate treatment. Behav. Brain Res. 210, 202–210. 10.1016/j.bbr.2010.02.032 20178820

[B49] Yochum C. L. Dowling P. Reuhl K. R. Wagner G. C. Ming X. (2008). VPA-induced apoptosis and behavioral deficits in neonatal mice. Brain Res. 1203, 126–132. 10.1016/j.brainres.2008.01.055 18316065

[B50] Zhang L. Chung S. K. Chow B. K. (2014). The knockout of secretin in cerebellar Purkinje cells impairs mouse motor coordination and motor learning. Neuropsychopharmacology 39, 1460–1468. 10.1038/npp.2013.344 24356714 PMC3988549

[B51] Zwaigenbaum L. Bryson S. Garon N. (2013). Early identification of autism spectrum disorders. Behav. Brain Res. 251, 133–146. 10.1016/j.bbr.2013.04.004 23588272

