# Supplementary material for: Aberrant Development and Synaptic Transmission of Cerebellar Cortex in a VPA Induced Mouse Autism Model
Source: Front Cell Neurosci. 2018 Dec 21;12:500. doi: 10.3389/fncel.2018.00500 (PMC6308145; doi:10.3389/fncel.2018.00500)
Supplement: Supplementary file 1 [file Data_Sheet_1.PDF]

Supplementary Information

Aberrant development and synaptic transmission of cerebellar cortex in a VPA induced mouse autism model

Ruanna Wang, Jiahui Tan, Junxiu Guo, Yuhan Zheng, Qing Han, Kwok-Fai So, Jiandong Yu and Li Zhang

Correspondence: Dr. Li Zhang (zhangli@jnu.edu.cn)

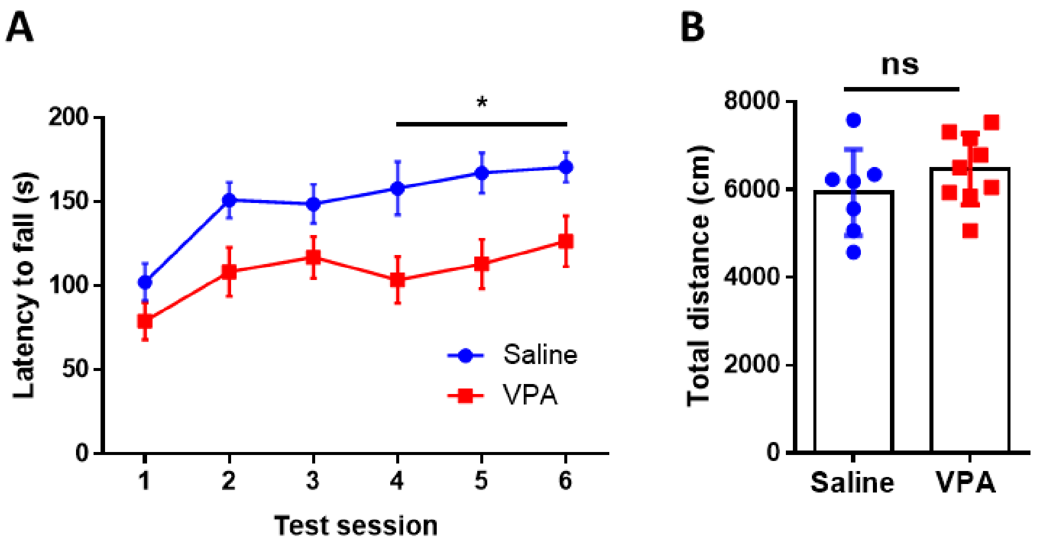

Supplementary Figure 1 Rota-rod motor learning and locomotor activity in an open-field of VPA mice. (A) Latency of mice on an accelerating rod across 6 daily train sessions. VPA mice showed similar performance as in saline control group at the beginning but presented less significant improvement with repeated training (2-way ANOVA with respect to treatment: $F_{(1,24)}$ = 8.755, *P*=0.0068; Bonferroni post-hoc comparison: *P*<0.05 at day 4 to day 6). *N*=12 mice in saline group, and 14 mice in VPA group. (B) Total distanced in a 15-min open field session was similar between VPA and control mice (2-sample *t*-test, t_{14} = 1.187, *P*=0.2550). ns, no significant difference; *, *P*<0.05; N=7 mice in saline group, and 9 mice in VPA group.

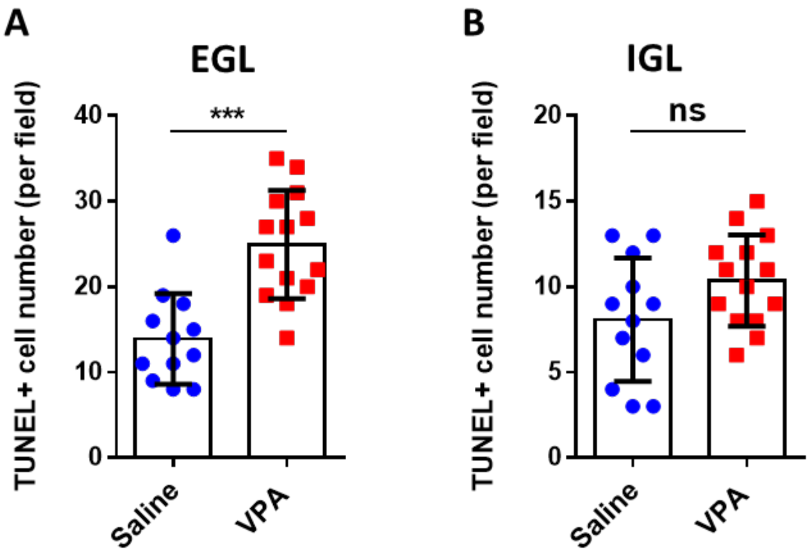

Supplementary Figure 2 Cell apoptosis in cerebellar cortex at P15. TUNEL assay revealed significantly higher apoptosis level in EGL of VPA mice comparing to saline control group (2-sample student *t*-test, $t_{24} = 4.766$, $P<0.0001$; A) but no significant difference when examining IGL (B). ns, no significant difference; ***, $P<0.0001$; N=12 slices from 4 mice in saline group, and 14 slices from 4 mice in VPA group.

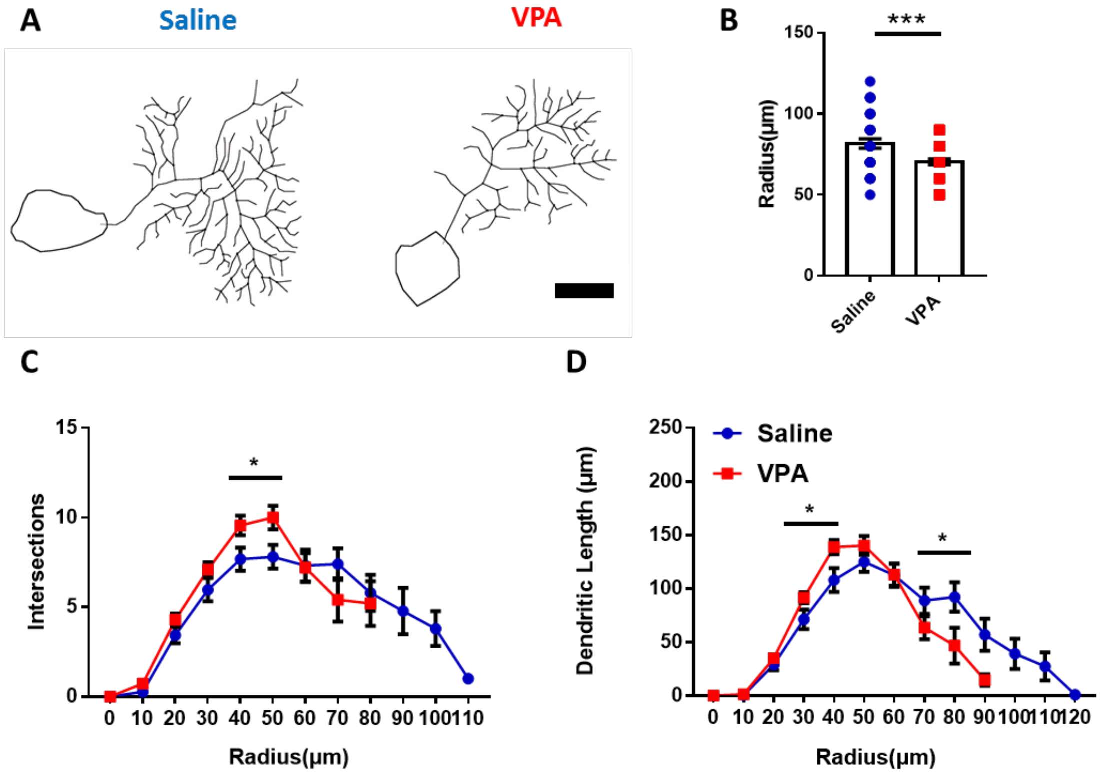

Supplementary Figure 3 Morphometry analysis of Purkinje cell at P9. (A) Representative dendrite plots of Purkinje cells after Golgi staining. (B) VPA mice had decreased radius of dendritic tree comparing to saline group (2-sample student *t*-test, t_{79}=3.462, *P*=0.0009). (C) Sholl analysis showed higher complexity of dendrite in VPA mice at 40~50μm segment (*P*<0.05 using multiple *t*-test). (D) VPA mice had longer dendrite branches in 30~40μm segment but shorter dendrite lengths in 70~80μm segment (*P*<0.05 using multiple *t*-test). *, *P*<0.05; ***, *P*<0.0001; N=38 cells from 4 mice in saline group, and 45 cells from 5 mice in VPA group. Scale bar, 50μm.

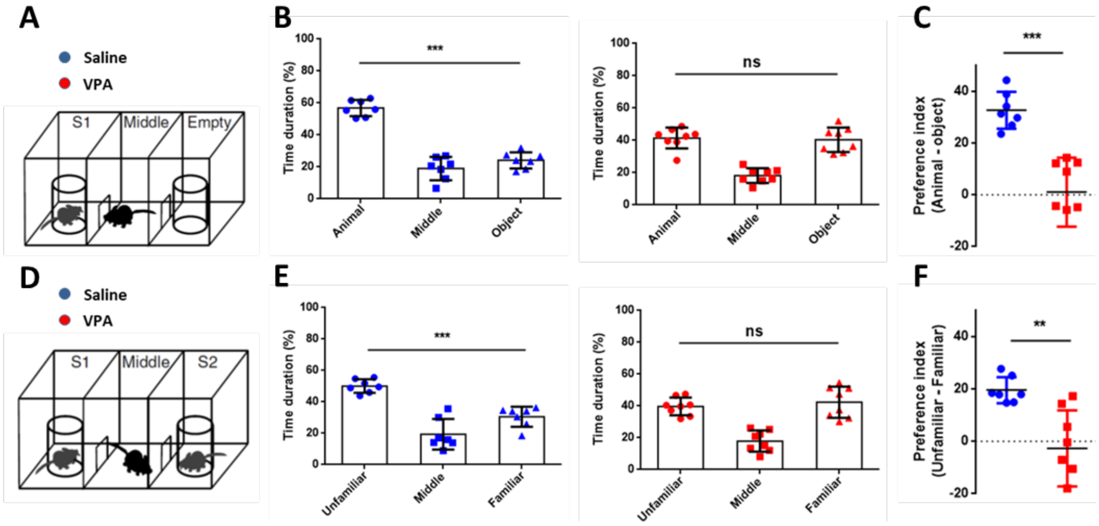

Supplementary Figure 4 Social deficits in VPA mice. (A) Schematic diagram for 3-chamber assay in testing sociability. (B) Quantitative analysis for social interaction time showed VPA mice spent similar time between animal and object (right) whilst control mice clearly displayed preference for animal over object (left). (C) Social preference index as calculated by the time difference between animal and object for each individual mouse. (D) Illustration for social novelty assay in 3-chamber apparatus. One unfamiliar mouse (S2) was placed in one side chamber, and the familiar mouse (S1) was placed in the other side. (E) Same as (B), showing similar preference for novel mice or familiar one in VPA group (right) whilst control group showed novel mouse preference (left). (F) Preference index as the difference between unfamiliar and familiar mouse interaction time. Ns, no significant difference; **, $P<0.01$; ***, $P<0.0001$ using one-way analysis of variance followed by Tukey post-hoc test (B and E) or 2-sampled t-test (C and F). $N=7$ for saline group and 8 for VPA group.

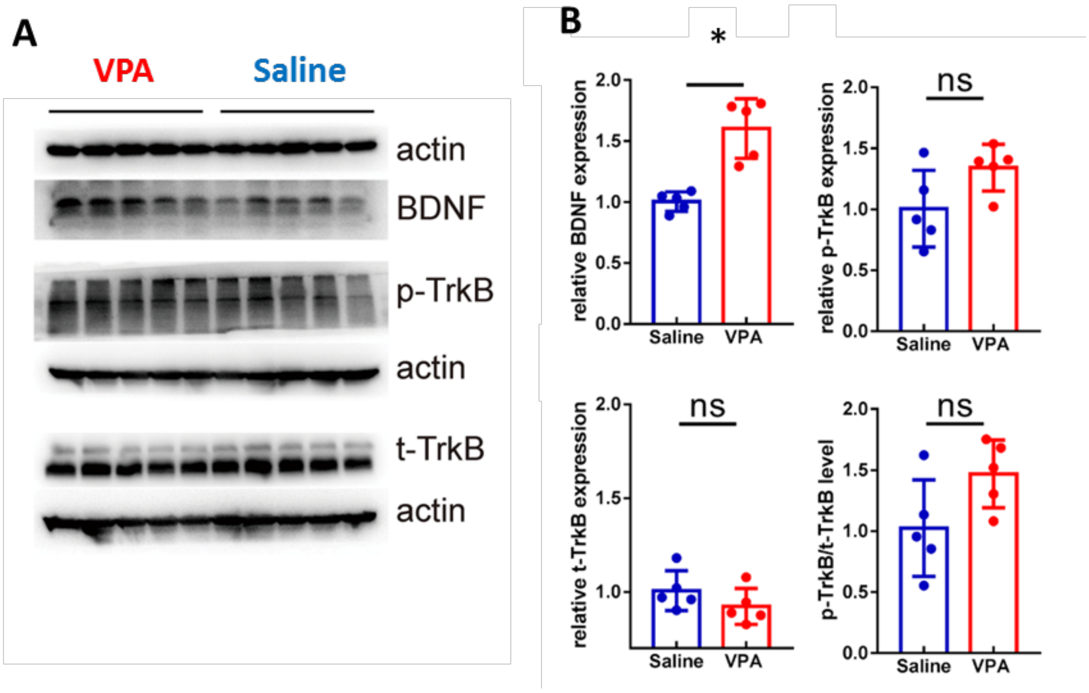

Supplementary Figure 5 Protein expression of BDNF-TrkB pathway. (A) Western blotting bands for BDNF, p-TrkB and t-TrkB proteins between VPA and saline groups. (B) Quantitative analysis for relative protein expression. VPA mice had significantly higher BDNF expression (2-sample student *t*-test, t_8=2.958, *P*=0.0182). No significant difference was found in p-TrkB, t-TrkB or p-TrkB/t-TrkB ratio (*P*>0.05). ns, no significant difference; *, *P*<0.05; N=5 mice in each group.
